# Inositol 1, 4, 5-trisphosphate-dependent nuclear calcium signals regulate angiogenesis and cell motility in triple negative breast cancer

**DOI:** 10.1371/journal.pone.0175041

**Published:** 2017-04-04

**Authors:** Erika Guimarães, Rodrigo Machado, Matheus de Castro Fonseca, Andressa França, Clarissa Carvalho, Ana Cândida Araújo e Silva, Brígida Almeida, Puebla Cassini, Bárbara Hissa, Luciana Drumond, Carlos Gonçalves, Gabriel Fernandes, Marina De Brot, Márcio Moraes, Lucíola Barcelos, José Miguel Ortega, André Oliveira, M. Fátima Leite

**Affiliations:** 1 Department of Molecular Medicine, Federal University of Minas Gerais, Belo Horizonte, Brazil; 2 Department of Physiology and Biophysics, Federal University of Minas Gerais, Belo Horizonte, Brazil; 3 Brazilian National Laboratory for Biosciences, Center for Research in Energy and Materials, Campinas, Brazil; 4 Department of Pharmacology, Federal University of Minas Gerais, Belo Horizonte, Brazil; 5 Department of Physics, Federal University of Minas Gerais, Belo Horizonte, Brazil; 6 Department of Biochemistry and Immunology, Federal University of Minas Gerais, Belo Horizonte, Brazil; 7 Genomics Sciences and Biotechnology of Universidade Católica de Brasília, Brasília, Brazil; 8 Department of Pathological Anatomy, Federal University of Minas Gerais, Belo Horizonte, Brazil; Rajiv Gandhi Centre for Biotechnology, INDIA

## Abstract

Increases in nuclear calcium concentration generate specific biological outcomes that differ from those resulting from increased cytoplasmic calcium. Nuclear calcium effects on tumor cell proliferation are widely appreciated; nevertheless, its involvement in other steps of tumor progression is not well understood. Therefore, we evaluated whether nuclear calcium is essential in other additional stages of tumor progression, including key steps associated with the formation of the primary tumor or with the metastatic cascade. We found that nuclear calcium buffering impaired 4T1 triple negative breast cancer growth not just by decreasing tumor cell proliferation, but also by enhancing tumor necrosis. Moreover, nuclear calcium regulates tumor angiogenesis through a mechanism that involves the upregulation of the anti-angiogenic C-X-C motif chemokine 10 (CXCL10-IP10). In addition, nuclear calcium buffering regulates breast tumor cell motility, culminating in less cell invasion, likely due to enhanced vinculin expression, a focal adhesion structural protein. Together, our results show that nuclear calcium is essential for triple breast cancer angiogenesis and cell migration and can be considered as a promising strategic target for triple negative breast cancer therapy.

## Introduction

Calcium (Ca^2+^) is a ubiquitous intracellular messenger responsible for controlling several cellular processes, including short duration events, such as contraction and secretion, as well as long-term responses such as gene transcription, proliferation and cell death [1]. Ca^2+^ signals are usually initiated by the binding of a hormone or a growth factor, originating a signaling cascade that produces diacylglycerol and inositol 1,4,5-trisphosphate (IP3). Subsequently, IP3 binds to its specific receptor (IP3R) on the membrane of the endoplasmic reticulum and triggers Ca^2+^ release. IPR3 can be found in the endoplasmic reticulum, in the nuclear envelope and in the nucleus along the nucleoplasmic reticulum [2]. Although Ca^2+^ signals can propagate throughout the cell, nuclear and cytosolic Ca^2+^ are regulated independently and can lead to distinct cell responses [3]. For instance, nuclear Ca^2+^ is well known to regulate gene transcription [4] and tumor cell proliferation *in vitro* and *in vivo* [5]. However, whether nuclear Ca^2+^ signaling regulates additional stages of tumor progression such as cell migration and angiogenesis remains unknown.

Breast cancer is the leading cause of death from cancer among women [6] and one of the most common cancers worldwide [7]. Among all breast cancer subtypes, the triple negative breast cancer (TNBC) subtype accounts for approximately 15–20% cases [8]. TNBC is defined as estrogen receptor (ER) negative, progesterone receptor (PR) negative and human epidermal growth factor receptor (HER2) negative. Due to its poor prognosis and survival rates TNBC is considered the most aggressive subtype among breast cancers [9]. Moreover, the lack of well-characterized molecular targets, broad spectrum chemotherapy remains the treatment of choice for TNBC, making this breast cancer subtype the most challenging to treat [9]. Indeed, even when responsive to therapy, TNBC tend to relapse and metastasize early [8]. These features make the search for new therapeutic targets urgently needed for TNBC.

The capability to treat cancer efficiently is directly related not only to the containment of the primary tumor, but also to the ability to interfere with the multistep metastatic process that involves events such as cell motility and cell migration [10, 11]. In this regard, antiangiogenic strategies represent an important antitumor target for cancer therapy [12, 13] since blood vessels are considered the principal exit route of metastatic cells from the primary tumor foci [14].

Here, we investigate whether buffering of nuclear Ca^2+^ regulates TNBC progression through modulation of angiogenesis and cellular migration. It is shown that decreased nuclear Ca^2+^ signaling reduced tumor blood vessel formation and consequentially prevented TNBC growth at least in part due to increased expression of CXCL10, a well-known angiostatic chemokine [15] and a promising target to prevent cancer metastasis [16]. In addition, we show that nuclear Ca^2+^ buffering reduced triple negative cancer cells motility and cell invasion associated with increased expression of vinculin, a focal adhesion protein that plays a central role in cell shape and motility [17]. Together our data demonstrate that nuclear Ca^2+^ is involved in several steps of TNBC growth and progression and constitutes a potential target for breast cancer treatment.

## Materials and methods

### Cell culture and animals

4T1 cells (American Type Culture Collection—ATCC), a widely used triple negative breast cancer cell line [18,19] and a murine model for stage IV human breast cancer, were grown at 37°C with 5% CO_2_ in RPMI-1640 supplemented with 10% fetal bovine serum (FBS), 100 units/mL penicillin and 50 g/mL streptomycin, all from GIBCO (Grand Island, NY). MDA-MB-231 and MDA-MB-468, also triple negative breast cancer cell lines, however, derived from human breast tumors, were purchased from ATCC and cultivated at 37°C with 5% CO_2_ in DMEM medium supplemented with 10% FBS, 100 units/mL penicillin and 50 g/mL streptomycin. HEK293 cells, used for adenoviral expansion, were grown at 37°C with 5% CO_2_ in DMEM supplemented with 10% FBS, 100 units/mL penicillin and 50 g/mL streptomycin, all from GIBCO (Grand Island, NY). Female Balb/C mice at 7–8 weeks (approximately 25-30g) were used for *in vivo* experiments. Animal care and experimental procedures were complied with Universidade Federal de Minas Gerais—Comissão de Ética no Uso de Animais (CEUA)—institutional animal welfare established guidelines (CEUA/UFMG/Approval 88/2013). Animals were maintained on a standard diet and water access *ad libitum* and housed under a 12-hour light–dark cycle.

### Nuclear IP3 buffering constructs

A recombinant adenovirus construct containing the binding region of the human type I IP3R, plus three nuclear localization sequences (NLS) and the monomeric red fluorescent protein (RFP) was used (IP3-sponge-NLS). The IP3-sponge-NLS buffers nuclear IP3, prevents IP3 binding to the nuclear IP3R and consequently blocks nuclear Ca^2+^ increase, as previously described for different growth factors, such as HGF and insulin [20–22]. For control, a similar adenovirus with either RFP alone or deleted IP3 gene (ΔIP3-NLS) with intact RFP and NLS sequences, to guarantee the nuclear localization of the construct, were used, as indicated. The adenoviral constructs were amplified using HEK293 and purified with Adenovirus Purification and Concentration Kit (Fast-Trap^®^, Millipore). For *in vitro* assays, control, ΔIP3-NLS and IP3-sponge-NLS viruses were used, except where noted. The adenoviruses (100–200 MOI) were applied to the cells 24, 48 or 72 hours prior to all experiments and infection rates were determined by analyzing RFP fluorescence and ranged from 80–90% of cells. In order to check the effectiveness of the infection we evaluated, the presence of RFP fluorescence in the cell nucleus and the percentage of Ca2+ increase in control, IP3-sponge-NLS and ΔIP3-NLS conditions upon EGF stimulation.

### Nuclear Ca^2+^ measurements

Ca^2+^ transients were monitored as previously described [5, 20, 23]. For these studies, 4T1, MDA-MB-231 and MDA-MB-468 cells were plated on 22x22cm coverslips placed on 6 well dish and 48 hours after adenovirus infection cells were incubated with 6μM fluo-4/AM (Invitrogen), for 30 minutes at 37°C and then imaged by time-lapse confocal microscopy for Ca^2+^ studies. Briefly, coverslips were transferred to a custom-built perfusion chamber and changes in fluo-4/AM fluorescence were measured after stimulation with epidermal growth factor (EGF), (200ng/mL; Sigma) on a ZEISS LSM 510 confocal microscope using a 63x, 1.4NA objective lens. Changes in fluorescence were normalized by the initial fluorescence (baseline) and expressed as fluorescence/ baseline fluorescence × 100% [23].

### Experimental tumor model

4T1 were inoculated subcutaneously (1 x 10^5^ cells/animal) and at days 5 and 10 after inoculation, tumors were injected with either adenoviral IP3-sponge-NLS or vehicle saline solution. After 15 days of tumor cell inoculation, animals were euthanized for tumor removal. Tumors were photographed, weighted and measured using a caliper ruler and tumor volume was assessed using the equation: tumor volume (mm^3^) = (lenght×width^2^)/2 [5].

### Magnetic Resonance Imaging (MRI)

MRI was performed as previously described [24]. Briefly, mice were anesthetized with halothane (4% induction, 1.5% maintenance) and oxygen (1.5 l/min) delivered by a facemask in a head holder to minimize artifact movements [25] and tumor images were obtained using 1mm slices on a 4.7 tesla MRI scanner. Multi-slice spin echo images in sequential coronal section were acquired 5, 10 and 15 days post tumor implantation. MeVisLab was used for image processing and tumor volume analyses were obtained after definition of tumor region of interest (ROI) using a MATLAB program.

### Tumor perfusion index

Tumors were monitored using noninvasive scanning laser-Doppler perfusion imaging (MoorLDPI-2, Moor Instruments). Images were collected 5, 10 and 20 days after tumor induction and tumor perfusion index was evaluated using moorLDI V5.0 software in regions of interest (ROI) within the tumor area.

### Immunohistochemistry

Identification of blood vessels and tumor cell proliferation was performed by staining with monoclonal antibodies against von Willebrand Factor, (DAKO) and Proliferating Cell Nuclear Antigen (PCNA) respectively. Sections from control and nuclear IP3-buffered tumors were fixed in 10% neutral buffered formalin and embedded in paraffin. Sections of 5μm were dewaxed and antigen retrieval was performed in citrate buffer containing 0.6% hydrogen peroxide. Nonspecific binding was blocked with 10% normal goat serum. Then, tumor sections were immunostained with von Willebrand Factor (1:40; DAKO) or PCNA antibodies (1:50; DAKO) for 1 hour at room temperature. After washing in Tris–HCl buffer, sections were incubated for 30 min at room temperature with the byotinylated Link Universal Streptavidin-HRP (DAKO). The reactions were revealed by applying 3,3′-diaminobenzidine in chromogenic solution (DAB), (DAKO). Sections were counterstained with hematoxylin and mounted in Permount (Fisher Scientific). Controls in which primary antibodies were omitted showed no specific staining. Histological images were obtained on a micro-camera (Olympus Q-color 5) coupled to a light microscope (Olympus BX43), captured with a plan-apochromatic objective (20x) and analyzed with Image-Pro Plus 4.5 (Media Cybernetics). Random tumor fields were evaluated for tumor vessel density and results are shown as number of blood vessel per tumor area. To evaluate proliferation pattern, tumor fields were randomly selected and a binary image was created to perform automatic quantification of positive PCNA nuclei. To evaluate tumor profile, slices were stained with anti-ER (1:50), anti-PR (1:200) and anti-HER2 (1:100) (Novacastra). After washing in Tris–HCl buffer, sections were incubated for 30 min at room temperature with the byotinylated Link Universal Streptavidin-HRP (DAKO). The reactions were revealed by applying 3,3′-diaminobenzidine in chromogenic solution (DAB), (DAKO)

### Tumor necrosis area

Tumors from both groups were fixed in 10% buffered formalin, and processed for paraffin embedding. 5μm sections were stained with hematoxylin and eosin and imaged on a stereoscopic microscope in low magnification. 5 tumor sections from 6 mice were randomly selected for morphological necrosis analysis. A region of interest, comprising the whole necrotic tissue, was delimited and the relative percentage of tumor necrotic area determined using ImageJ software. The necrotic area was distinguished by the absence of tumor cell nuclei, loss of tissue organization and the presence of abundant inflammatory infiltrates.

### Cellular proliferation assay

*In vitro* cell proliferation assay was assessed by manual counting incorporation as previously described [21].

### Transcriptome profiling

High-throughput mRNA sequencing (RNASeq) was used to monitor changes in gene expression after nuclear Ca^2+^ buffering. RNA isolation was performed using RNeasy (QIAGEN). Illumina RNAseq cDNAs libraries were prepared from 3μg of total RNA extracted from cells previously infected with IP3-sponge-NLS or control adenoviruses. The amplified library was sequenced on an Illumina HiSeqTM2500 (paired-end 75bp) at Yale Center for Genomic Analysis (YCGA, Yale University). Reads were mapped to the draft genome sequence of GRCm38.75, using bowtie2 aligner and standard parameters. Statistical analyses to determine differentially expressed genes were performed with an R program using the DESeq2 package and images were generated using R functions and libraries. Differentially expressed genes represent a fold change ≥2.

### Pathway construction

A text-mining approach was used to uncover associations between RNASeq upregulated and downregulated genes and angiogenesis. Articles whose abstracts represent the angiogenic process were manually selected and used as a training set on the web application Medline Ranker (http://cbdm-01.zdv.uni-mainz.de/~jfontain/cms/) against a test set comprising the whole PubMed (http://www.ncbi.nlm.nih.gov/pubmed) query “Angiogenesis”; articles were ranked on the test set according to the co-occurrence of terms on both sets abstracts [26], and top 1000 ranked articles (p<0.01) used as an input on PESCADOR web application to determine interactions between gene symbols found on the abstracts [27]. These gene symbols were crosschecked against the upregulated and downregulated genes on RNASeq. Forms of interactions between genes and angiogenesis were used as a reference for PathVisio software pathway construction [28].

### ELISA

Concentration of IP-10/CXCL10 in cell culture supernatants was determined by Quantikine Mouse CXCL10 immunoassay kit according to manufacture’s instructions (R&D Systems).

### Real time PCR

Total cellular RNAs were extracted using Trizol reagent (Sigma) according to the instructions of the user manual. cDNAs were generated from 1μg RNA using the High Capacity cDNA Reverse Transcription Kit (Life Technologies). Real-time PCR analyses were conducted with SYBR Green PCR Supermix (Bio-Rad) using PCR primers on a CFX96 Real Time PCR system (Bio-Rad). β-actin primer sequences were as follows: 5' GTGACGTTGACATCCGTAAAG 3' (forward) and 5' GCCGGACTCATCGTACTCC 3' (reverse); Mouse CXCL10 primer sequences were as follows: 5' CTCGCAAGGACGGTCCGCTG 3' (forward) and 5' CGTGGCAATGATCTCAACACGTGG 3' (reverse). Human CXCL10 primer sequences were as follows: 5’ GCCTCTAGACTGAGAATTCTGATAAACCC 3’ and 5’ CACCAAATCAGCTGCTACTA 3’ (reverse). Relative mRNA expression was determined by the comparative Ct method using Bio-Rad software (Bio-Rad).

### Cell migration

Cell migration was determined on a wound-healing assay for 4T1, MDA-MB-231 and MDA-MB-468 cells. Five random wound fields were captured 0, 24 and 48 hours after scratch wounding and closure was assessed by measurements of relative wound area. Hydroxyurea was present (1μM) to prevent potential proliferative effects in wound closure [29].

### Cell tracking

Wound healing was monitored using a BioStation IM-Q Time Lapse Imaging System (Nikon) and 4T1 cell tracking was performed using Volocity^®^ Software -Tracking in Volocity Software (Perkin Elmer). Cells were randomly selected for live cell tracking analyses and cell nucleus position identified in each frame in a 20 minute interval over 4 hours and subsequently connected by the built in tracking algorithm followed by quantification of average speed, distance and directionality, which was defined as displacement divided by the total distance, i.e. a cell moving in a straight line defined as 1.

### Defocusing microscopy

Defocusing microscopy technique was used to analyze cellular dynamics of 4T1 cells. In order to access modifications in both plasma membrane and cytoskeleton dynamics of control and nuclear Ca^2+^ buffered cells, cells of both groups were recorded for 30 minutes as previously described [30]. Experiments were performed using a Eclipse TI inverted microscope equipped with a 530 nm wavelength green filter, a stage-heated oil-immersion objective Nikon Apo Tirf 60X, NA 1.49 (Nikon), and an environmental chamber (Chamlide IC- CU:109, Live Cell Instrument). Images were captured using a 12bit Uniq camera (Epix Inc, Buffalo Grove) with gain of 11.75db and capture rate of 1 frame per second. Focal distance was controlled using a Nikon Perfect Focus System apparatus and gray levels calibration was performed as described [30]. Temporal autocorrelation was analyzed using ImageJ (NIH, Bethesda, MA, USA) plugins and adjusted in KaleidaGraph Software (Synergy Software, Essex Junction, VT, USA) using single exponential decay curves that carry information about their amplitude and time decay characteristics.

### Cell invasion assay

4T1 cell invasion through a Matrigel membrane was quantified using a QCM Cell Invasion Assay (Chemicon) according to the manufacturer’s instructions.

### Immunofluorescence

Vinculin immunofluorescence was performed using the Actin Cytoskeleton/Focal Adhesion Staining Kit (Millipore) according to the manufacturer’s instructions. 4T1 cells (2×10^5^ cells/well) were plated onto 6 wells plate containing 22×22 glass coverslips and 24 hours later infected with the IP3-sponge-NLS adenovirus. Control (non-infected) and nuclear IP3-buffered cells were then fixed with 4% paraformaldehyde (Electron Microscopy Science) and permeabilized with 0.5% Triton^®^ X100 (Sigma). After washing in Phosphate Buffered Saline (PBS) (Sigma), unspecific binding was blocked using PBS, 10% BSA, 10%, Triton^®^ 0.5% and 5% goat serum for 1 hour at room temperature. Immunolabeling with primary vinculin monoclonal antibody (1:200; Millipore) was performed for 1 hour at room temperature, followed by 1-hour incubation at room temperature with goat anti-mouse secondary antibody conjugated with Alexa Fluor 488 (1:500; Life Technologies) and DAPI (1:1000; Millipore) for nuclei staining. Controls in which primary antibodies were omitted showed no specific staining. Images were obtained using a Zeiss LSM 510 confocal microscope (Thornwood, NY, USA). Twenty cells were analyzed with ImageJ Analyze Particles plug-in to quantify the number of vinculin positive structures and average area of vinculin staining.

### Immunoblottings

4T1 cells were harvested as described and protein content was quantified using Bradford assay [20]. Whole cell protein lysate (20μg) were separated by 12% SDS-PAGE (BIORAD). Blots were incubated for 2 hours at room temperature with primary anti-vinculin monoclonal antibody (Millipore) at 1:5000. After washing, blots were incubated in HRP-conjugated goat anti-mouse IgG secondary antibody, 1:5000 at room temperature for 1 hour. β-actin was used as housekeeping gene and blots were incubated for 2 hours at room temperature with the primary anti-β-actin antibody (Millipore) at 1:5000 dilution. Immunodetection was carried out using enhanced chemiluminescence (ImageQuant LAS 4000, GE Healthcare Lifesciences) and quantification was performed using ImageJ software.

### Statistical analyses

All data presented represent at least three independent experiments and are expressed as mean ± SD. Statistical analysis was performed using Prism software (GraphPad 5.0, San Diego, CA). Statistical analyses between multiple groups were performed with ONE-way Anova and between two groups using Student's t test with p values < 0.05 considered statistically significant.

Real time PCR experiments were evaluated by Mann Whitney test, with p values < 0.05 considered statistically significant.

## Results

### Nuclear Ca^2+^ is essential for TNBC growth *in vitro* and *in vivo*

**Fig 1:**
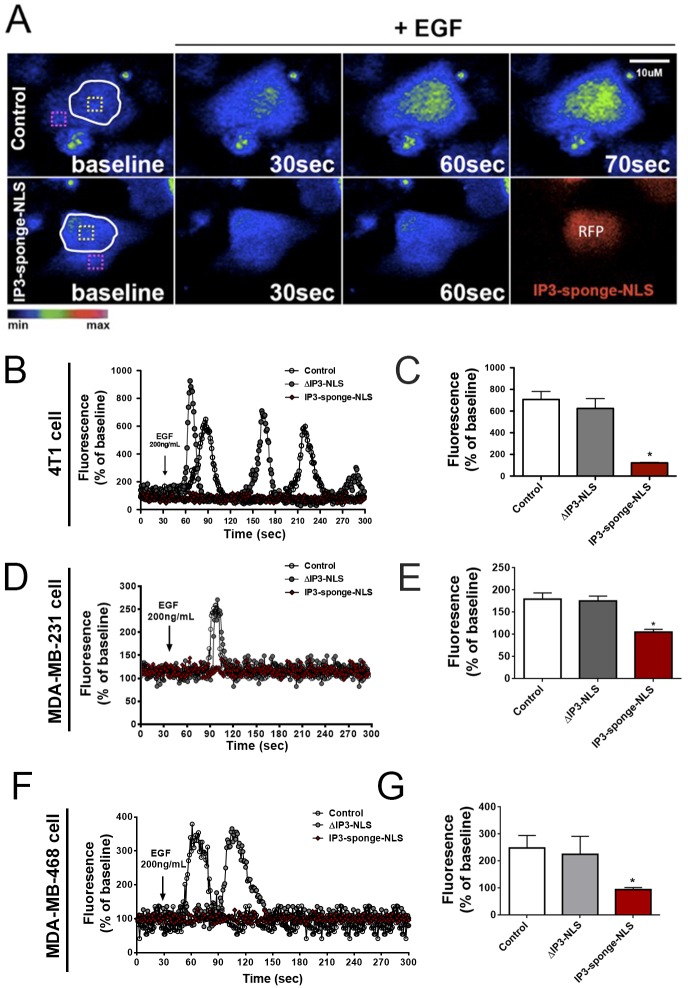
Nuclear IP3 buffering decreases Ca+2 signals in the nucleus of triple negative breast cancer cells. (A) Confocal serial images of control (top panels) or IP3-sponge-NLS infected 4T1 cells (bottom panels), loaded with fluo-4/AM and stimulated with 200ng/ml EGF for 30, 60 and 70 seconds. Dashed yellow boxes represent the nuclear region of interest. Images were pseudocolored according to the scale shown at the bottom. Scale bar = 10μM. (B, D and F) Representative time course of nuclear fluorescence levels of 4T1, MDA-MB-231 and MDA-MB-468 cells, respectively. Black arrow indicates initial EGF stimulation and fluorescence level is expressed as % of basal fluorescence. (C, E and G) Average nuclear fluorescence peak (n = 20 cells) of each cell group and condition throughout the time-course; fluorescence level is expressed as % of basal fluorescence. * = p <0.05 versus control. Values are expressed as mean ± SD.

**Fig 2:**
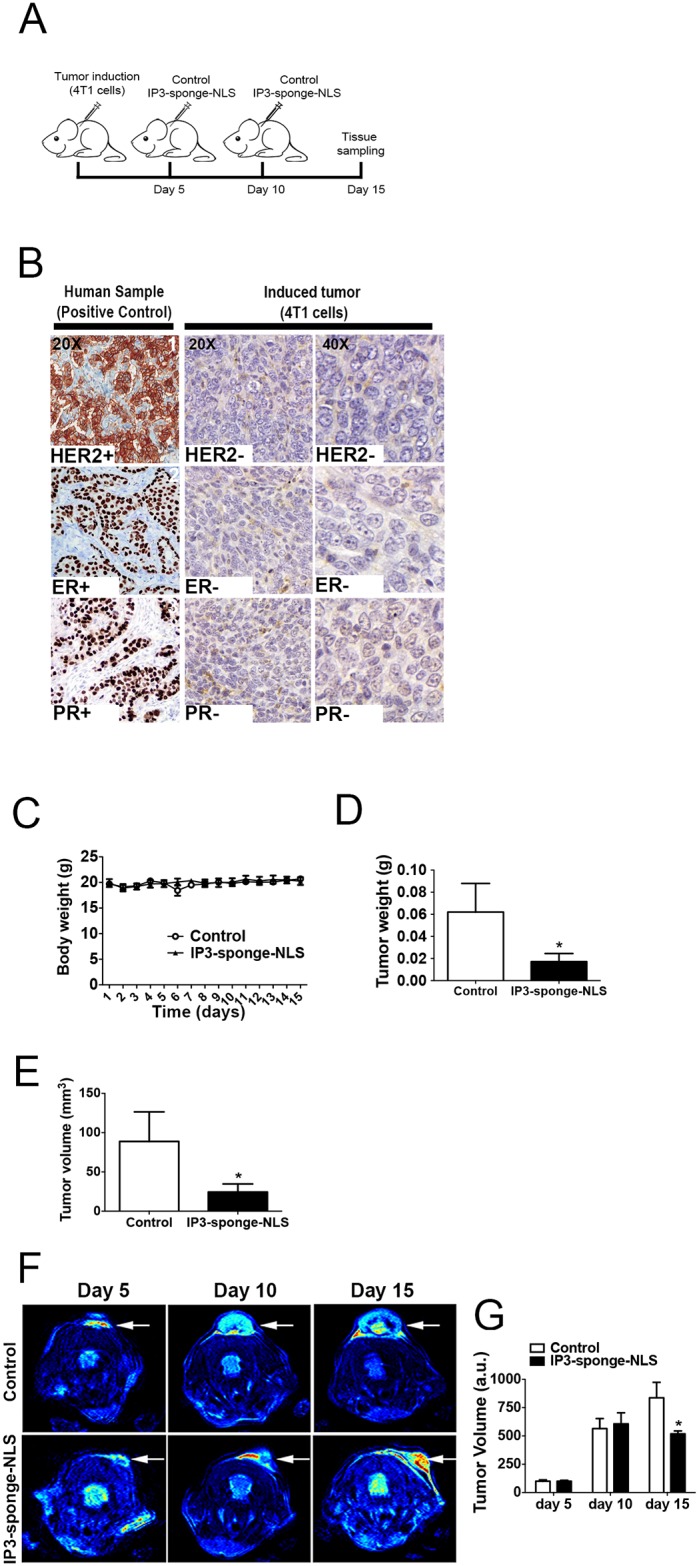
Nuclear IP3 buffering impairs TNBC growth in vivo. (A) In vivo experimental design. (B) Representative photomicrographs of human invasive breast carcinomas positive for HER2 (1), ER (2) and PR (3) that were used as external control in our immunohistochemical study (left panels) (20x magnification). Right panels show that 4T1-induced tumor has no staining for any of the markers used (20x and 40x magnification). (C) Daily weight along the experiment (n = 5 animals). (D) Tumor weight at day 15, after 2 sequential treatments with either the IP3-sponge-NLS construct or vehicle solution (n = 5). (E) Tumor volume at day 15, after the 2 sequential treatments (n = 5 animals). (F) Representative images of tumors magnetic resonance imaging, at days 5, 10 and 15 after tumor induction. White arrows indicate tumor location. (G) Quantification of MRI tumor volume at indicated days after tumor induction (n = 5 animals). * = p <0.05 versus control. Values are expressed as mean ± SD.

It is known that nuclear Ca^2+^ signaling plays an important role in cell cycle progression and proliferation in several cell types [2, 5, 20, 21]. In order to investigate whether nuclear Ca^2+^ is also involved in breast tumor growth, we first evaluated the contribution of nuclear Ca^2+^ signals to the growth of TNBC. First, subcellular Ca^2+^ signals were evaluated in different triple negative breast cancer cell lines expressing a nuclear IP3 buffering adenovirus construct (IP3-sponge-NLS), which was shown to selectively reduce Ca^2+^ signals in the nucleus [20–22]. Using time-lapse confocal microscopy, Ca^2+^ transients in the nucleus of 4T1, MDA-MB-231 and MDA-MB-468 cells were measured upon stimulation with EGF, a growth factor that induces a rapid increase in calcium levels in the nucleus and cytosol of several cell types through IP3 generation [31], (Fig 1A–1G). We found that in all breast cancer cell lines analyzed, IP3-sponge-NLS nearly abolished nuclear Ca^2+^ signals induced by EGF (4T1: control = 707 ± 73%; IP3-sponge-NLS = 179 ± 13%; MDA-MB-231: control = 179 ± 3.5%; IP3-sponge-NLS = 105 ± 1.5%; MDA-MB-468: control = 248 ± 12%; IP3-sponge-NLS = 94 ± 2%; n = 55 cells for each condition), (Fig 1C, 1E and 1G). No change in Ca^2+^ fluorescence level was observed throughout the time courses analyzed of the IP3-sponge-NLS infected cells. On the other hand, the adenovirus ΔIP3-NLS, with intact NLS and RFP sequences, but no IP3 binding region, was unable to prevent EGF-induced nuclear Ca^2+^ increase in all cell lines (4T1: control = 707 ± 73%; ΔIP3-NLS = 624 ± 60%; MDA-MB-231: control = 179 ± 3.5%; ΔIP3-NLS = 175 ± 2%; MDA-MB-468: control = 248 ± 12%; ΔIP3-NLS = 337 ± 18; n = 55 cells for each condition) (Fig 1C, 1E and 1G). The peak Ca^2+^ amplitude observed in cells infected with ΔIP3-NLS was similar to control group. Next, we investigated whether nuclear Ca^2+^ regulates breast tumor growth *in vivo*. 4T1 cells, a mouse cell line and, therefore, suitable for the *in vivo* experiments, were implanted subcutaneously in mice and after tumor establishment, tumors were directly injected with either the adenovirus that buffers nuclear IP3 or vehicle saline solution (Fig 2A). Red dots indicate IP3-Sponge-NLS infected cells (S1 Fig). In order to check if the tumor developed was indeed triple negative as the cells injected, immunohistochemistry labeling was performed for HER2, RE and RP receptors. Images collected showed no labeling for any of the receptors. Human breast cancer samples were used as a positive control (Fig 2B). Local administration of the IP3-sponge-NLS was not accompanied by animal weight loss (Fig 2C), an indication that the adenoviral treatment did not cause significant side effects. Both tumor mass (control = 0.06 ± 0.02 g; IP3-sponge-NLS = 0.01 ± 0.007 g, n = 5, p<0.05), (Fig 2D), and tumor volume (control = 89 ± 38 mm^3^; IP3-sponge-NLS = 24 ± 10 mm^3,^ n = 5, p<0.05), (Fig 2E) were reduced in animals treated with IP3-sponge-NLS when compared to control mice 15 days after tumor induction. Indeed, sequential measurements of tumor volume by MRI showed that nuclear IP3 buffering impaired 4T1 breast tumor growth (control = 837 ± 13 a.u.; IP3-sponge-NLS = 519 ± 25 a.u., n = 5, p<0.05), (Fig 2F and 2G). These results show that nuclear Ca^2+^ regulates TNBC growth *in vivo*.

**Fig 3:**
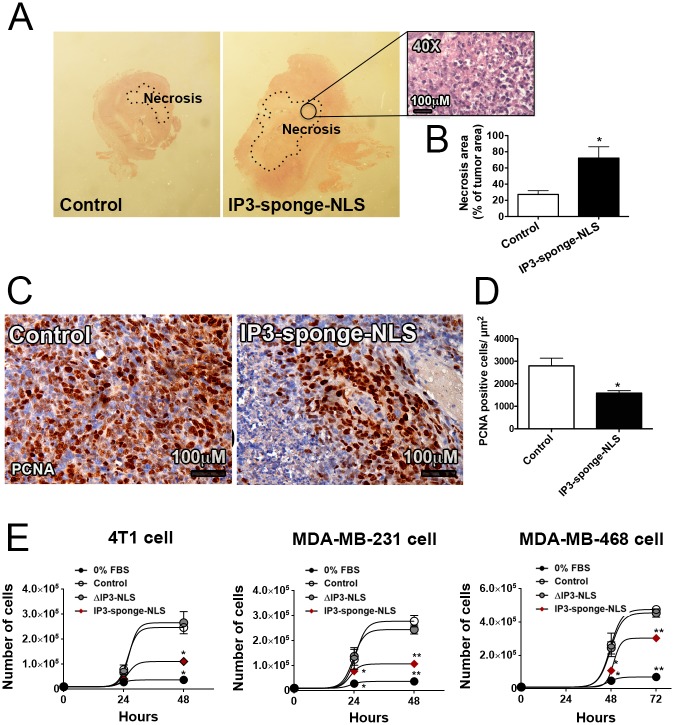
Nuclear IP3 buffering regulates necrosis and proliferation. (A) Stereoscopic microscope representative image of tumor necrosis area (dashed lines), 15 days post tumor induction. Image in detail represents the tumor necrotic area (40× magnification). Scale bar = 100μM. (B) Average tumor necrosis area as % of total tumor area. Results are representative of 6 animals per group and 5 randomly selected fields of view per animal. * = p <0.05 versus control. Values are expressed as mean ± SD. (C) Representative image of PCNA immunohistochemistry; scale bar = 100μM. (D) PCNA quantification by average number of PCNA positive cells/μm2. Results are representative of 6 animals/group and 5 randomly selected fields of view/animal. * = p <0.05. Values are expressed as mean ± SD. (E) Cell growth assay performed with 4T1 (left panel), MDA-MB-231 (middle panel) and MDA-MB-468 cells at 0, 24 and 48 hours of culture in control, ΔIP3-NLS and IP3-sponge-NLS groups. Triplicate in 3 individual experiments was performed. 0% fetal bovine serum (0%) group represents the experimental negative control. n = 3 individual experiment per group and condition; * and ** = p <0.05 * = p <0.05 versus control. Values are expressed as mean ± SD.

To further evaluate the mechanism by which nuclear IP3 buffering could cause breast tumor suppression *in vivo*, tumor histopathology was assessed. Examination of tumor sections revealed that relative tumor necrosis area was increased in IP3-sponge-NLS-treated group compared with control group (control = 27 ± 5%; IP3-sponge-NLS = 72 ± 15%, n = 6, p<0.05), (Fig 3A and 3B). Distribution of IP3-Sponge-NLS cells in the tumor is shown on S1 Fig. Moreover, *in vivo* nuclear IP3 buffering also suppressed tumor cell proliferation as shown by the reduced number of PCNA positive cells per μm^2^ subjected to IP3-sponge-NLS treatment (control = 2796 ± 338 cells; IP3-sponge-NLS = 1583 ± 112 cells), (Fig 3C and 3D). Similar results were obtained by *in vitro* cell growth assay performed not only with 4T1 cells (Fig 3E, left panel) but also MDA-MB-231 (Fig 3E, middle panel) and MDA-MB-468 (Fig 3E, right panel) cells, in which cells infected with IP3-sponge-NLS also proliferated less than control and ΔIP3-NLS infected cells (4T1 48hs: 0% FBS = 0.3 ± 0.1 x 10^5^, control = 2.7 ± 0.3 x 10^5^; ΔIP3-NLS = 2.95 ± 0.3 x 10^5^; IP3-sponge-NLS = 1.1± 0.1 x 10^5^; MDA-MB-231 48hs: 0% FBS = 0.4 ± 0.01 x 10^5^, Control = 2.8 ± 0.1 x 10^5^, ΔIP3-NLS: 2 ± 0.3 x 10^5^; IP3-sponge-NLS: 0.9 ± 0.1 x 10^5^; MDA-MB-468 72hs: 0% FBS = 0.7 ± 0.03 x 10^5^, control = 4.7 ± 1.2 x 10^5^; ΔIP3-NLS = 4.5 ± 1.7 x 10^5^; IP3-sponge-NLS = 3 ± 0.03 x 10^5^) (Fig 3E; S1 Table). MDA-MB-468 cells were evaluated after 48 and 72 hours since it presented a reduced infection level only with 24 hours of incubation with any of the viruses. Together, these findings show that buffering nuclear Ca^2+^ prevents TNBC growth, both by decreasing tumor cell proliferation and by enhancing tumor necrotic area.

### Nuclear Ca^2+^ regulates tumor angiogenesis

**Fig 4:**
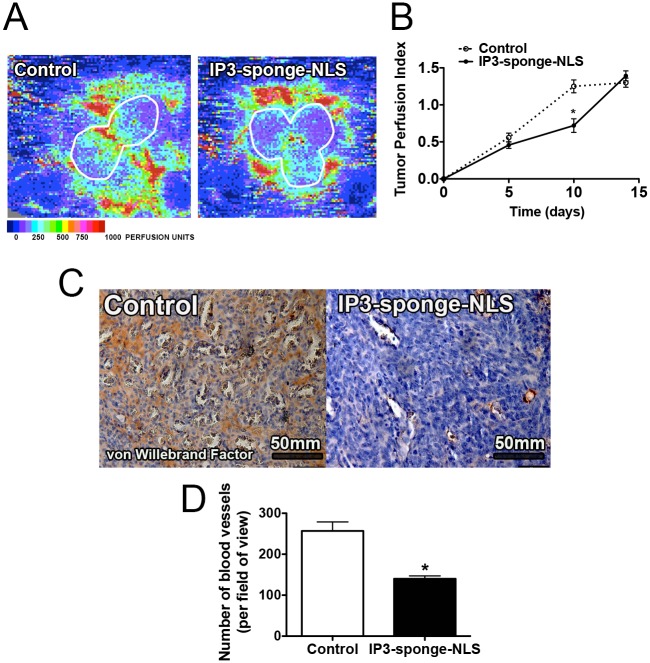
Nuclear IP3 buffering reduces TNBC angiogenesis. (A) Representative image of non-invasive Laser-Doppler 10 days after tumor induction. White lines indicate tumor region of interest. Tumor perfusion index is assembled from mean Doppler velocity and analyzed according to the color scale at the bottom. (B) Average tumor perfusion index 5, 10 and 15 days after tumor induction (n = 5, for each time point). (C) Representative image of von Willebrand factor immunostaining. Scale bar = 50mm. (D) Average number of blood vessels per field of view. Results are average values of 6 animals per group and 5 randomly selected fields of view per animal. * = p <0.05 versus control. Values are expressed as mean ± SD.

Since tumor necrosis can result from decreased vascularization [32], we investigated whether buffering nuclear Ca^2+^ could affect blood vessel formation as well as tumor blood flow. A non-invasive Doppler flowmeter was used for sequential assessment of breast tumor perfusion. Doppler images demonstrated lower blood perfusion in tumors treated with the IP3-sponge-NLS adenovirus in comparison with control tumors (Day 10: control = 1.0 ± 0.2 a.u.; IP3-sponge-NLS = 0.6 ± 0.07 a.u, n = 5, p<0.05), (Fig 4A and 4B). Since reduction of the blood perfusion index typically indicates loss of tumor blood vessels, blood vessel immunohistochemical staining was used to assess tumor vascular density. Nuclear IP3 buffering caused a reduction in the number of tumor vessels (control = 257 ± 22 vessels; IP3-sponge-NLS = 140 ± 7 vessels, n = 6, p<0.05), (Fig 4C and 4D). These results are in agreement with previous findings showing that angiogenesis is a limiting factor in tumor expansion [13, 33]. Additionally, our data show that nuclear Ca^2+^ plays a crucial role in blood vessel formation in the tumor microenvironment.

### Nuclear Ca^2+^ alters the expression of angiogenic related genes

**Fig 5:**
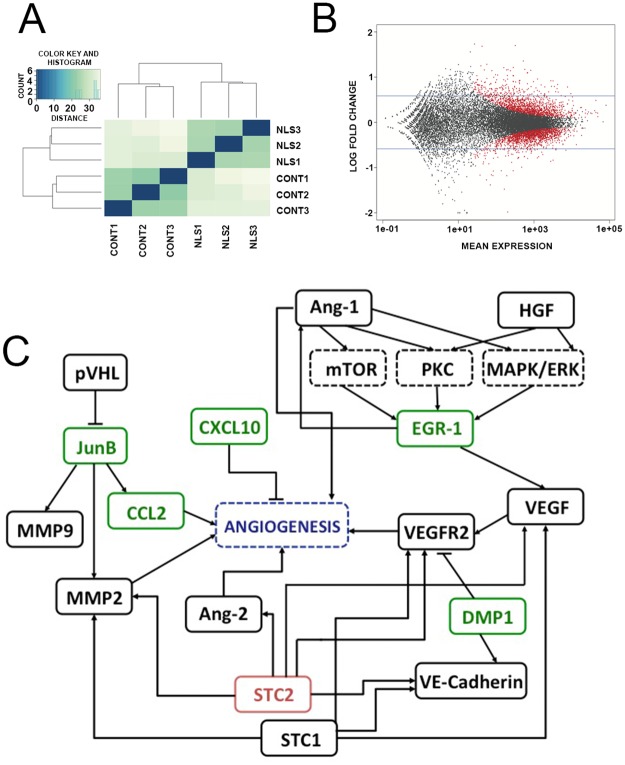
Nuclear IP3 buffering alters the expression of angiogenic related genes. (A) Euclidian distances between samples of both groups: control (CONT1, CONT2 and CONT3 samples) and IP3-sponge-NLS (NLS1, NLS2 and NLS3 samples). (B) Detection of genes differentially expressed. Blue horizontal lines represent the fold change expression cut off (2x fold change or 1 in Log2 scale). Each point represents a single gene of the reference transcriptome. The red points correspond to genes significantly differentially expressed (p<0.01 versus control). (C) Representative pathway indicates indirect existing relationships between differentially expressed genes that are both regulated by nuclear Ca+2 and involved in angiogenic processes. Rectangles represent genes products; green rectangles indicate upregulated genes and red rectangles, downregulated genes. Arrowheads represent activation and T-bars represent repression. Black dashed rectangles indicate signaling pathways and blue dashed lines represent the concept of angiogenesis.

**Fig 6:**
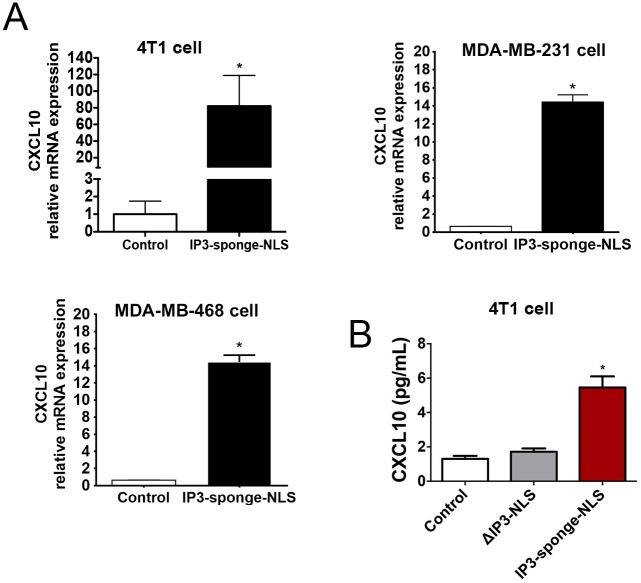
Nuclear IP3 buffering alters the expression and secretion of the angiostatic CXCL10. (A) CXCL10 mRNA relative expression levels evaluated in 4T1 (upper left panel), MDA-MB-231 (upper right panel) and MDA-MB-468 cells (lower left panel). (B) CXCL10 secreted levels of 4T1 cells. n = 3 individual experiments. * = p <0.05 versus control. Values are expressed as mean ± SD.

Nuclear Ca^2+^ is known to regulate gene expression [21, 34–36]. Ca^2+^ signaling can affect gene transcription, directly by binding to DNA [37] or indirectly by modulating transcription factors activity [35]. To understand the mechanism by which nuclear Ca^2+^ might regulate tumor angiogenesis, we analyzed whole cell transcriptome profile using an Illumina-based RNASeq approach. We found that IP3-sponge-NLS treatment altered the gene expression profile in 4T1 breast tumor cells (Fig 5A): 82 genes were identified as significantly downregulated and 204 genes identified as upregulated (p<0.01; fold change>2), (Fig 5B). A full list and description of these genes can be found in S2 Table. From the pool of significantly regulated genes identified, we selected those ones involved in angiogenesis through a bioinformatic text-mining approach for further analysis. Thereafter, forms of interactions between genes and angiogenesis were used as a reference for a pathway construction [27, 28]. A subset of 6 genes regulated by nuclear Ca^2+^ buffering and that are known to play a significant role in angiogenesis, were detected (Fig 5C). The 5 upregulated genes were: Jun-B Proto-Oncogene (JUNB), C-C motif chemokine 2 (CCL2), C-X-C motif chemokine 10 (CXCL10), Dentin matrix protein (DMP1), Early growth response protein 1 (EGR1), represented in green and 1 downregulated gene: Stanniocalcin 2 (STC2), represented in red. Since the chemokine CXCL10 is a well-known angiostatic factor [38] we validated the expression analysis result by quantitative real time PCR and showed that CXCL10 mRNA levels are enhanced after nuclear IP3 buffering not only in 4T1 cells (Upper left panel, control = 1.00 ± 0.75 a.u.; IP3-sponge-NLS = 82 ± 36 a.u.), but also in MDA-MB-231 (Upper right panel, control = 0.5 ± 0.2 a.u.; IP3-sponge-NLS = 11 ± 0.6 a.u.) and 468 cell lines (Lower left panel, control = 0.6 ± 0.02 a.u., IP3-sponge-NLS = 11.3 ± 0.5 a.u.) (Fig 6A). Moreover, secretion of CXCL10 was increased in supernatants of 4T1 breast tumor cells in which nuclear IP3 was buffered, but it was unaffected in ΔIP3-NLS infected cells (control = 1.3 ± 0.2 pg/mL; ΔIP3-NLS = 1.7 ± 0.2 pg/mL; IP3-sponge-NLS = 5.4 ± 0.6 pg/mL (Fig 6B). Together, these results show that tumor angiogenesis is regulated by nuclear Ca^2+^ likely through an increase in CXCL10 expression and secretion.

### Nuclear Ca^2+^ regulates cell motility

**Fig 7:**
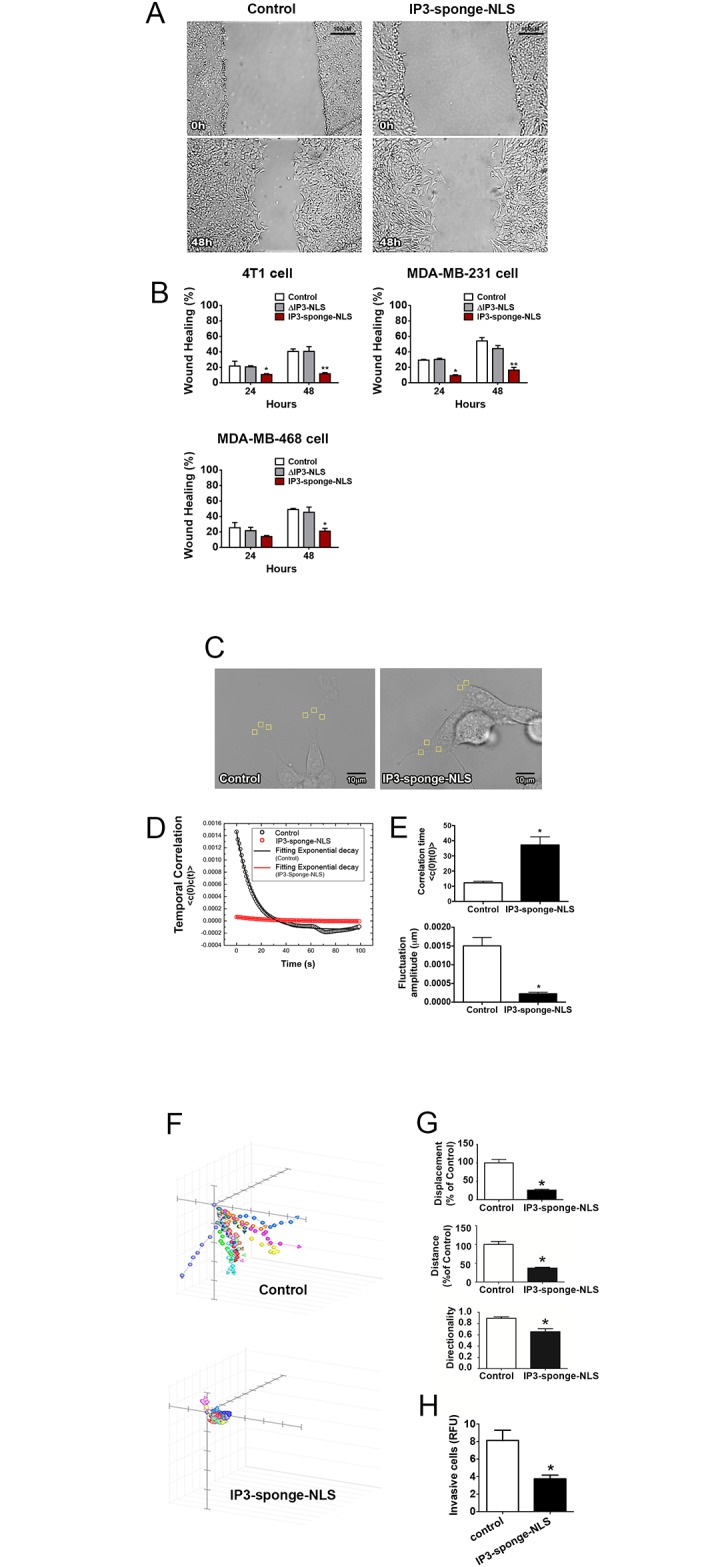
Nuclear IP3 buffering reduces breast cancer cells motility. (A) Representative image of in vitro wound healing assay performed with 4T1 cells. Images were selected from a representative well 48 hours after nuclear IP3 buffering. Scale bar = 100μM. (B) Average of wound healing closure 24 and 48 hours after nuclear IP3 buffering (n = 5 wells/group, for each time point). Results represent % of initial wound area (0 hours). (C) Bright field representative image of defocusing microscopy experiment performed with 4T1 cells. Yellow squares represent area of temporal correlation analyses. Scale bar = 10μm. (D) Correlation temporal function (<c(0)t(0)>) representative graph. (E) Average correlation time values. Results are average values of 3 areas per cell and 3 cells per group. Average fluctuation amplitude values. Results are average values of 10 areas per cell and 3 cells per group. (F) Tracking of individual 4T1 cells. Migratory cells, located at the limit of the wound, were selected and nuclear cell position was assessed in 20 minutes intervals during 4 hours (n = 20 cells/group). (G) Average displacement (upper panel), traveled distance (middle panel) and directionality (lower panel) in each group. Data are shown as % of control. (H) Average 4T1 cell invasion through Matrigel in both experimental groups; data showed by relative fluorescence units (RFU)(n = 3 individual experiments/group). * = p <0.05 versus control. Values are expressed as mean ± SD.

**Fig 8:**
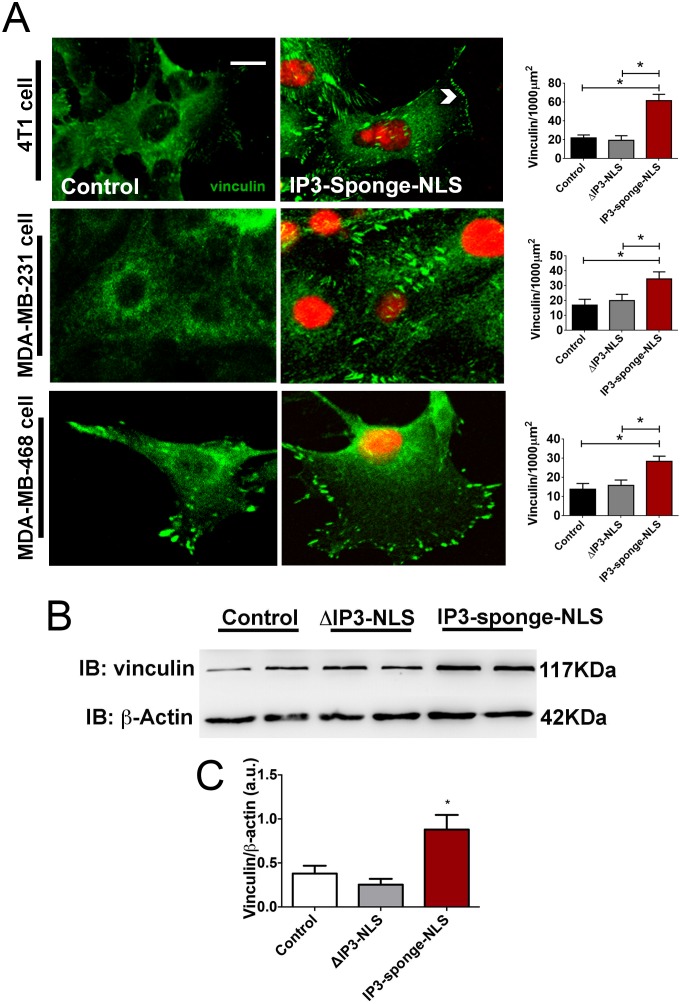
Nuclear IP3 buffering increases breast tumor expression of vinculin. (A) Representative immunofluorescence images of 4T1 (upper panels), MDA-MB-231 (middle panels) and MDA-MB-468 control and IP3-Sponge-NLS-infected cells (lower panels) labeled with specific anti-vinculin (green) antibody and nuclear IP3-sponge-NLS infection (red). Scale bar = 10μm. Average number of vinculin–positive regions for the cell types analyzed are shown as vinculin/1000μm2 on each respective graph (n = 20 cells/group). (B) Representative immunoblottings of total 4T1 cell lysates probed with anti-vinculin and anti-βactin, used as protein loading control. (C) Immunoblottings densitometry analysis. Results show β-actin normalized proteins expression (n = 3 individual experiments/group). * = p <0.05 versus control. Values are expressed as mean ± SD.

Despite the implication of primary tumor establishment in the final outcome of the disease, tumor metastasis is also considered a key event in the prognosis and overall survival in cancer. The ability of tumor cells to undergo migration and invasion allows for dissemination from the primary tumor site to lymph nodes and blood stream and colonization of distant organs [39, 40]. To assess the role of nuclear Ca^2+^ on cell migration, a wound-healing assay was used and quantification of wound closure rate showed that nuclear IP3 buffering diminishes 4T1, MDA-MB-231 and MDA-MB-468 cells migration (Fig 7A and 7B), such that buffered cells were unable to efficiently occupy the wound gap (4T1–24 hours: control = 29.5 ± 0.5%; ΔIP3-NLS = 26.7 ± 0.1%; IP3-sponge-NLS = 9.3± 0.6%; 48 hours: control = 50 ± 3%; IP3-sponge-NLS = 38 ± 2%; MDA-MB-231- 24 hours: control = 21.7 ± 3.5%; ΔIP3-NLS = 20.7 ± 0.7%; IP3-sponge-NLS = 10.7± 0.7%; 48 hours: control = 47 ± 6.6%; ΔIP3-NLS = 33.7± 7.3%; IP3-sponge-NLS = 11 ± 0.9%; MDA-MB-468-24 hours: control = 21.5 ± 1.5%; ΔIP3-NLS = 21.8 ± 2.6%; IP3-sponge-NLS = 14± 1%; 48 hours: control = 42.7 ± 6.3%; ΔIP3-NLS = 45.3± 4%; IP3-sponge-NLS = 21 ± 2%). Additionally, defocusing microscopy technique was used to analyze cellular dynamics of 4T1 cells and differences in cortical fluctuations between control and nuclear IP3 buffered cells. Nuclear IP3 buffered cells showed longer relaxation times (control = 12 ± 1s; IP3-sponge-NLS = 37 ± 5s) and smaller fluctuation amplitudes (control = 15 ± 9 x 10^−4^ μm; IP3-sponge-NLS = 2 ± 1 x 10^−5^ μm), (Fig 7C–7E) which indicate that nuclear IP3 buffering turned cells stiffer and less motile. Furthermore, cell movement was evaluated by tracking the motion of individual 4T1 cells at the migration wound front (Fig 7F). Quantification of these movement tracks revealed that nuclear IP3 buffered cells moved a shorter distance, exhibited reduced displacement and reduced directionality of cell locomotion compared to control cells (Fig 7G). Since nuclear Ca^2+^ negatively regulated cell motility, we next investigated whether nuclear IP3 buffering also modulates cell invasion. We found that in addition to its role in cell migration, nuclear Ca^2+^ also reduces cell invasion, as shown on the assay performed with 4T1 cells (control = 8 ± 1 a.u.; IP3-sponge-NLS = 4 ± 0.4 a.u.), (Fig 7H). To better understand the potential mechanism by which nuclear Ca^2+^ regulates cell migration, a crucial step for cell invasion, we evaluated the pattern of vinculin expression, since cells with reduced expression of vinculin become less adherent and more motile [41]. Moreover, vinculin is a critical component in the regulation of focal adhesion stability [42]. For that reason, vinculin-deficient cells are less stiff than normal cells and exercise lower traction forces [43, 44]. Indeed, we found that IP3 buffering increased the number of subcellular vinculin–positive regions in 4T1 cell line (control = 29 ± 5; ΔIP3-NLS = 19 ± 4 and IP3-sponge-NLS = 64 ± 3). We also validated this information in human TNBC cell lines MDA-MB-231 (control = 16 ± 5; ΔIP3-NLS = 19 ± 5 and IP3-sponge-NLS = 34 ± 6) and MDA-MB-468 (control = 13 ± 5; ΔIP3-NLS = 16 ± 5 and IP3-sponge-NLS = 28 ± 4) (Fig 8A). Accordingly, vinculin expression was increased in nuclear IP3 buffered 4T1 cells and it was unaffected in ΔIP3-NLS cells (Fig 8B and 8C) (control = 0.35 ± 0.1 a.u.; ΔIP3-NLS = 0.25 ± 0.02 a.u.; IP3-sponge-NLS = 0.9 ± 0.2 a.u.). Together, these results show that nuclear Ca^2+^ regulates several steps of the cell motility cycle in breast tumors.

## Discussion

Despite evident progress in therapeutic strategies in a variety of tumors, breast cancer remains a deadly disease [7]. Among breast cancer subtypes, TNBC shows the most aggressive nature, with higher rates of relapse and shorter overall survival, what makes the development of targetable and innovative therapeutic approaches extremely urgent. Ongoing preclinical and clinical efforts are focused on developing more refined drug targets to control breast cancer, which include a wide range of antiangiogenic treatments [33, 45]. However, in TNBC, the most promising antiangiogenic strategies remain controversial and no major impact in the overall patient survival has been efficiently demonstrated [46, 47]. This might result from the activation of compensatory routes. Therefore, the development of a multi-target drug that regulates more than one stage of tumor progression may be desirable to maximize the efficacy and to minimize therapeutic resistance during TNBC treatment. In the current work, nuclear Ca^2+^ was evaluated as a possible target for the development of new therapies to arrest TNBC progression. We demonstrated that nuclear Ca^2+^ regulates TNBC tumor expansion, due to its role in several well-known signaling pathways that are crucial for tumor growth and maintenance, including [1] growth, by decreasing cell proliferation and increasing tumor necrotic area; [2] angiogenesis, by disrupting tumor blood flow and [3] migration, by decreasing tumor cell motility. More studies are still needed to translate our current findings into therapeutic strategies; however, the antitumor effects of nuclear IP3 buffering point nuclear Ca^2+^ as a tangible target for further cancer therapy studies.

Considerable experimental evidence has been implicated nuclear Ca^2+^ in the regulation of cancer cell proliferation [2, 5, 21, 34]. For instance, studies performed in hepatocellular carcinoma cells [5] and in human squamous carcinomas cells [21], showed that nuclear Ca^2+^ buffering, either alone or in association with radiation, reduced cell proliferation. Our current data corroborate and expand these findings by showing that nuclear Ca^2+^ also prevents TNBC growth *in vivo*. Although the precise mechanism by which nuclear Ca^2+^ regulates TNBC cell proliferation is still not fully understood, it has been previously shown that nuclear Ca^2+^ buffering can stop cell cycle progression by synchronizing tumor cells at prophase [5]. Moreover, nuclear Ca^2+^ can modulate the promoter region activity of genes involved in cell proliferation [34] and it can prevent the upregulation of the tyrosine kinase receptor and the metalloproteinase expression induced by irradiation [21], two well-established activators of tumor cell growth.

Tumor angiogenesis depends on a delicate balance between angiogenic and anti-angiogenic factors. Few pro-angiogenesis associated genes were up-regulated by IP3-sponge-NLS as well as some anti-angiogenic factors. However, the IP3s-ponge-NLS outcome in tumors was inhibition of angiogenesis and vessel formation. In this case, the angiogenic imbalance observed here, occurs probably through the robust CXCL10 upregulation effects that overcame the pro-angiogenic factors likewise regulated by nuclear Ca^+2^ buffering. We evaluated the expression levels of CXCL10, the most relevant anti-antiangiogenic factor on tumor growth, since the main outcome is angiogenesis abrogation. We have demonstrated that nuclear Ca^2+^ buffering promotes CXCL10 upregulation. Interestingly, the expression of several pro-angiogenic related genes was altered by nuclear Ca^2+^ buffering, such as the CCL2 chemokine and the EGR1 transcription factor. However, the upregulation of pro-angiogenic genes under the IP3-sponge-NLS treatment, which might indicate a compensatory tumor behavior, is not able to counteract the angiostatic effect of the observed CXCL10 overexpression; the only gene found in our pathway analysis associated with tumor angiogenesis blockage. This hypothesis is supported by previous findings that show CXCL10 as a potent angiostatic factor, which inhibit a number of angiogenic activities, including human tumor-derived angiogenesis [48, 49] that can induce the dissociation of newly formed blood vessels even in the presence of angiogenic factors [50].

TNBC is likely to spread beyond the breast [8], the most fearsome aspect of cancer. For that, tumor cells must scape from the primary tumor and invade another tissue [51]. Although it is known that intracellular Ca^2+^ signaling promotes the directional cell migration [52], our current data show a specific role for nuclear Ca^2+^ in cancer cell motility. We found that nuclear Ca^2+^ buffered-cells presented an unpolarized and ineffective movement that is similar to the one described in motionless cells [53]. The reduced migratory capacity observed here could be a consequence of the altered cell cortical fluctuations. Indeed, as shown by defocusing microscopy, the increment in the relaxation time constant in nuclear-Ca^2+^-buffered cells and the associated reduction of amplitude of those fluctuations, indicate impairment in the ability to form membrane ruffles. The reduction of membrane fluctuations caused by nuclear Ca^+2^ buffering may turn cells more rigid and less motile [30]. In addition, these effects in cell migration and invasion might be a result of alterations in vinculin expression. Several studies showed that depletion of vinculin renders cells less adherent and more motile, implying a worse prognosis in several types of tumors, including breast cancer [54].

Indeed, vinculin-deficient cells have reduced stress fiber formation, less focal adhesion formation and inhibition of cell protrusions [17, 55]. The alterations in vinculin expression described here could lead strongly adherent cells in which cell protrusions formation could be impaired, preventing cell invasion, as well as the series of consecutive attachment and detachment events that composes the migration cycle.

Together, our results provide compelling evidence for nuclear Ca^2+^ involvement in tumor growth, especially due to the newly reported role of nuclear Ca^2+^ in angiogenesis and cell migration, important steps of tumor progression. Therefore, nuclear Ca^2+^ buffering might be considered as a potent target for TNBC treatment, since it controls not only the primary tumor cell proliferation but also several steps of the tumor growth cascade.

## Supporting information

S1 Fig Infection pattern of IP3-sponge-NLS in 4T1 breast tumors. Three representative confocal images of three individually generated tumors treated according to the designed protocol (Fig 2A). Red dots indicate 4T1 infected cells. Scale bar: 100μM. 40x magnification.
(TIF)

S1 Table TNBC cell growth after nuclear Ca^2+^ buffering. Values of cell growth assay performed with 4T1 (left panel), MDA-MB-231 (middle panel) and MDA-MB-468 cells at 0, 24 and 48 hours of culture in control, ΔIP3-NLS and IP3-sponge-NLS groups. Triplicate in 3 individual experiments was performed. 0% fetal bovine serum (0%) group represents the experimental negative control. n = 3 individual experiment per group and condition; Values are expressed as mean ± SD.
(PDF)

S2 Table List of genes upregulated or downregulated after nuclear Ca^2+^ buffering. List of genes with altered expression caused by nuclear calcium reduction. Ensembl Code: Ensembl reference gene code (http://www.ensembl.org/index.html); log2 FoldChange: average gene expression; Gene: Gene nomenclature code; Description: gene function description the nomenclature.
(PDF)
