# Supplementary figures and images for: Inositol 1, 4, 5-trisphosphate-dependent nuclear calcium signals regulate angiogenesis and cell motility in triple negative breast cancer
Source: PLoS One. 2017 Apr 4;12(4):e0175041. doi: 10.1371/journal.pone.0175041 (PMC5380351; doi:10.1371/journal.pone.0175041)

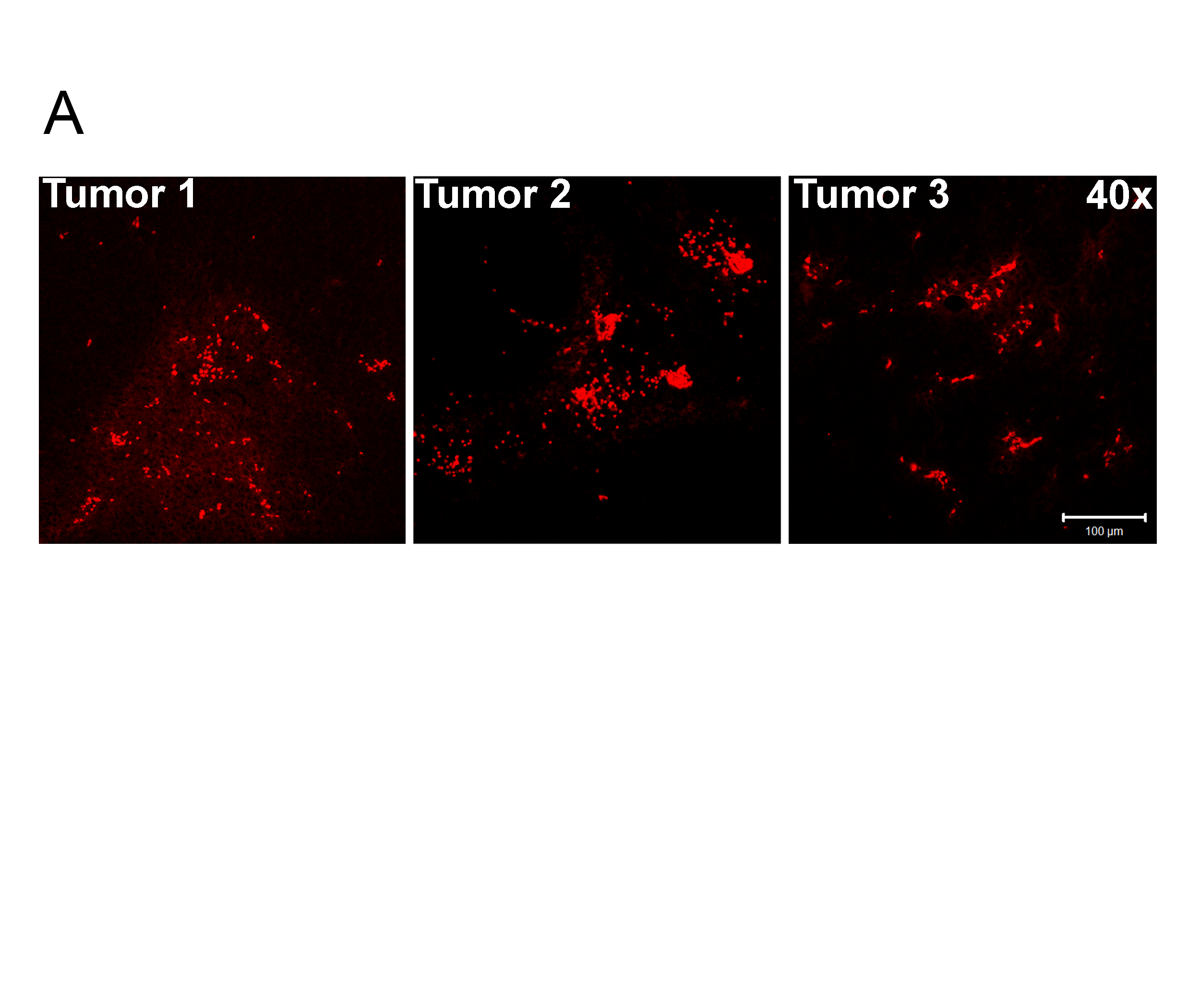

Supplement: S1 Fig — Three representative confocal images of three individually generated tumors treated according to the designed protocol (Fig 2A). Red dots indicate 4T1 infected cells. Scale bar: 100μM. 40x magnification. (TIF) [file pone.0175041.s001.tif]
