# Supplementary material for: Inositol 1, 4, 5-trisphosphate-dependent nuclear calcium signals regulate angiogenesis and cell motility in triple negative breast cancer
Source: PLoS One. 2017 Apr 4;12(4):e0175041. doi: 10.1371/journal.pone.0175041 (PMC5380351; doi:10.1371/journal.pone.0175041)
Supplement: S1 Table — Values of cell growth assay performed with 4T1 (left panel), MDA-MB-231 (middle panel) and MDA-MB-468 cells at 0, 24 and 48 hours of culture in control, ΔIP3-NLS and IP3-sponge-NLS groups. Triplicate in 3 individual experiments was performed. 0% fetal bovine serum (0%) group represents the experimental negative control. n = 3 individual experiment per group and condition; Values are expressed as mean ± SD. (PDF) [file pone.0175041.s002.pdf]

4T1 cells

	0 hours	24 hours	48 hours
**0 % FBS**	0.1×10^5	$0.2 \pm 0.1 \times 10^5$	$0.3 \pm 0.1 \times 10^5$
**Control**	0.1×10^5	$0.9 \pm 0.1 \times 10^5$	$2.7 \pm 0.3 \times 10^5$
**ΔIP3-NLS**	0.1×10^5	$0.9 \pm 0.2 \times 10^5$	$2.95 \pm 0.3 \times 10^5$
**IP3-sponge-NLS**	0.1×10^5	$0.3 \pm 0.1 \times 10^5$	$1.1 \pm 0.1 \times 10^5$

MDA-MB-231

	0 hours	24 hours	48 hours
**0 % FBS**	0.1×10^5	$0.4 \pm 0.04 \times 10^5$	$0.4 \pm 0.01 \times 10^5$
**Control**	0.1×10^5	$1.4 \pm 0.3 \times 10^5$	$2.8 \pm 0.1 \times 10^5$
**ΔIP3-NLS**	0.1×10^5	$1.3 \pm 0.2 \times 10^5$	$2 \pm 0.3 \times 10^5$
**IP3-sponge-NLS**	0.1×10^5	$0.7 \pm 0.08 \times 10^5$	$0.9 \pm 0.1 \times 10^5$

MDA-MB-468

	0 hours	48 hours	72 hours
**0 % FBS**	0.1×10^5	$0.4 \pm 0.04 \times 10^5$	$0.7 \pm 0.03 \times 10^5$
**Control**	0.1×10^5	$2.6 \pm 0.4 \times 10^5$	$4.7 \pm 1.2 \times 10^5$
**ΔIP3-NLS**	0.1×10^5	$2.4 \pm 0.2 \times 10^5$	$4.5 \pm 1.7 \times 10^5$
**IP3-sponge-NLS**	0.1×10^5	$1 \pm 0.5 \times 10^5$	$3 \pm 0.03 \times 10^5$
