# Supplementary material for: Inositol 1, 4, 5-trisphosphate-dependent nuclear calcium signals regulate angiogenesis and cell motility in triple negative breast cancer
Source: PLoS One. 2017 Apr 4;12(4):e0175041. doi: 10.1371/journal.pone.0175041 (PMC5380351; doi:10.1371/journal.pone.0175041)
Supplement: S2 Table — List of genes with altered expression caused by nuclear calcium reduction. Ensembl Code: Ensembl reference gene code (http://www.ensembl.org/index.html); log2 FoldChange: average gene expression; Gene: Gene nomenclature code; Description: gene function description the nomenclature. (PDF) [file pone.0175041.s003.pdf]

Table 1. List of genes upregulated or dowregulated after nuclear calcium buffering				
**Ensembl Code**	**log2 FoldChange**	**Gene**	**Description**	**Gene Expression**
ENSMUSG00000000290	0,628133041	Itgb2	Integrin beta-2	Upregulated
ENSMUSG00000000627	1,032335318	Sema4f	Semaphorin-4F	Upregulated
ENSMUSG00000000791	0,652204631	Il12rb1	Interleukin-12 receptor; subunit beta-1	Upregulated
ENSMUSG00000001604	0,970185422	Tcea3	Transcription elongation factor A; protein 3	Upregulated
ENSMUSG00000003617	0,792793158	Cp	Ceruloplasmin	Upregulated
ENSMUSG00000005951	0,709229586	Shpk	Sedoheptulokinase	Upregulated
ENSMUSG00000005986	0,775239141	Ankrd13d	Ankyrin repeat domain-containing protein 13 D	Upregulated
ENSMUSG00000006403	0,589854915	Adamts4	A disintegrin-like and metallopeptidase, thrombospondin 4	Upregulated
ENSMUSG00000007039	0,65740248	Ddah2	Dimethylarginine dimethylaminohydrolase 2	Upregulated
ENSMUSG00000009145	0,746846955	Dqx1	ATP-dependent RNA helicase DQX1	Upregulated
ENSMUSG00000012428	0,608841204	Steap4	STEAP family member 4	Upregulated
ENSMUSG00000017390	0,667777492	Aldoc	Fructose-bisphosphate aldolase C	Upregulated
ENSMUSG00000017830	0,906806503	Dhx58	DEXH (Asp-Glu-X-His) box polypeptide 58	Upregulated
ENSMUSG00000017897	0,913716614	Eya2	Eyes absent 2 homolog (Drosophila)	Upregulated
ENSMUSG00000018916	1,158777374	Csf2	Colony stimulating factor 2 (granulocyte-macrophage)	Upregulated
ENSMUSG00000019102	0,732985103	Aldh3a1	Aldehyde dehydrogenase family 3, subfamily A1	Upregulated
ENSMUSG00000020034	0,942371306	Tcp11l2	T-complex 11 (mouse) like 2	Upregulated
ENSMUSG00000020159	0,765017506	Gabrp	Gamma-aminobutyric acid (GABA) A receptor, pi	Upregulated
ENSMUSG00000020638	1,165140369	Cmpk2	Cytidine monophosphate (UMP-CMP) kinase 2, mitochondrial	Upregulated
ENSMUSG00000020641	1,150003618	Rsad2	Radical S-adenosyl methionine domain containing 2	Upregulated
ENSMUSG00000020865	0,747807143	Abcc3	ATP-binding cassette, sub-family C (CFTR/MRP), member 3	Upregulated
ENSMUSG00000021025	0,622576087	Nfkbia	NF-kappa-B inhibitor alpha (I-kappa-B-alpha) (IkB-alpha)	Upregulated
ENSMUSG00000021200	1,283190961	Asb2	Ankyrin repeat and SOCS box-containing 2	Upregulated
ENSMUSG00000021250	0,744330117	Fos	Proto-oncogene c-Fos (cellular oncogene fos)	Upregulated
ENSMUSG00000021792	0,687143748	Fam213a	Redox-regulatory protein FAM213A	Upregulated
ENSMUSG00000021876	0,617300923	Rnase4	Ribonuclease 4 (Ribonuclease, RNase A family 4)	Upregulated
ENSMUSG00000021903	1,212385572	Galnt15	Polypeptide N-acetylgalactosaminyltransferase 15	Upregulated

ENSMUSG00000021950	0,644060821	Anxa8	Annexin A8	Upregulated
ENSMUSG00000022037	0,670249459	Clu	Clusterin	Upregulated
ENSMUSG00000022126	1,033118703	Irg1	Immunoresponsive gene 1	Upregulated
ENSMUSG00000022159	0,62322765	Rab2b	RAB2B, member RAS oncogene family	Upregulated
ENSMUSG00000022425	1,045678748	Enpp2	Ectonucleotide pyrophosphatase/phosphodiesterase 2	Upregulated
ENSMUSG00000022773	0,744839736	Ypel1	Yippee-like 1 (Drosophila)	Upregulated
ENSMUSG00000023186	0,828255772	Vwa5a	von Willebrand factor A domain containing; 5A	Upregulated
ENSMUSG00000024063	0,607916178	Lbh	Protein LBH (Limb bud and heart-expressed protein)	Upregulated
ENSMUSG00000024164	0,743797332	C3	Complement component 3	Upregulated
ENSMUSG00000024299	0,695223207	Adamts10	A disintegrin-like and metallopeptidase, thrombospondin 10	Upregulated
ENSMUSG00000024381	0,707296079	Bin1	Bridging integrator 1	Upregulated
ENSMUSG00000025492	0,598473289	Ifitm3	Interferon induced transmembrane protein 3	Upregulated
ENSMUSG00000025498	1,140236052	Irf7	Interferon regulatory factor 7	Upregulated
ENSMUSG00000025887	0,701521795	Casp12	Caspase 12	Upregulated
ENSMUSG00000025888	0,671498561	Casp1	Caspase 1	Upregulated
ENSMUSG00000025921	0,76177749	Rdh10	Retinol dehydrogenase 10	Upregulated
ENSMUSG00000026222	0,715693093	Sp100	Nuclear antigen Sp100	Upregulated
ENSMUSG00000026259	1,159547953	Ngef	Neuronal guanine nucleotide exchange fator	Upregulated
ENSMUSG00000026463	0,820385752	Atp2b4	ATPase, Ca++ transporting, plasma membrane 4	Upregulated
ENSMUSG00000026535	0,597516833	Ak1	Interferon activated gene 202B	Upregulated
ENSMUSG00000026822	0,917102276	Lcn2	Neutrophil gelatinase-associated lipocalin	Upregulated
ENSMUSG00000027175	0,608322661	Tcp11l1	T-complex 11 like 1	Upregulated
ENSMUSG00000027247	0,777921959	Arhgap1	Rho GTPase activating protein 1	Upregulated
ENSMUSG00000027399	0,63263777	Il1a	Interleukin-1 alpha (IL-1 alpha)	Upregulated
ENSMUSG00000027456	0,648981668	Sdcbp2	Syndecan binding protein (syntenin) 2	Upregulated
ENSMUSG00000027514	1,03485901	Zbp1	Z-DNA binding protein 1	Upregulated
ENSMUSG00000028015	0,586811032	Ctso	Cathepsin O	Upregulated
ENSMUSG00000028037	1,209764117	Ifi44	Interferon-induced protein 44	Upregulated
ENSMUSG00000028268	1,029062937	Gbp3	Guanylate binding protein 3	Upregulated

ENSMUSG00000028269	1,09204039	Gbp1	Guanylate binding protein 2, interferon-inducible	Upregulated
ENSMUSG00000028270	1,100159143	Gbp2	Interferon-induced guanylate-binding protein 2	Upregulated
ENSMUSG00000028793	0,673119194	Rnf19b	Ring finger protein 19B	Upregulated
ENSMUSG00000029019	0,620792504	Nppb	Natriuretic peptide type B	Upregulated
ENSMUSG00000029093	0,649085593	Sorcs2	Sortilin-related VPS10 domain containing receptor 2	Upregulated
ENSMUSG00000029154	0,619598426	Cwh43	PGAP2-interacting protein	Upregulated
ENSMUSG00000029298	0,924472319	Gbp9	Guanylate-binding protein 9	Upregulated
ENSMUSG00000029304	0,78018225	Spp1	Secreted phosphoprotein 1	Upregulated
ENSMUSG00000029307	0,889144372	Dmp1	Dentin matrix protein 1	Upregulated
ENSMUSG00000029380	0,677652925	Cxcl1	Chemokine (C-X-C motif) ligand 1	Upregulated
ENSMUSG00000029561	0,957239425	Oasl2	2'-5' oligoadenylate synthetase-like 2	Upregulated
ENSMUSG00000029771	0,596077957	Irf5	Interferon regulatory factor 5	Upregulated
ENSMUSG00000029869	0,840100945	Ephb6	Ephrin type-B receptor 6	Upregulated
ENSMUSG00000030102	0,900497388	Itpr1	Inositol 1,4,5-trisphosphate receptor 1	Upregulated
ENSMUSG00000030107	0,668756341	Usp18	Ubiquitin specific peptidase 18	Upregulated
ENSMUSG00000030630	0,816317046	Fah	Fumarylacetoacetate hydrolase	Upregulated
ENSMUSG00000030921	0,955656956	Trim30a	Tripartite motif-containing 30A	Upregulated
ENSMUSG00000030966	0,641860414	Trim21	Tripartite motif-containing 21	Upregulated
ENSMUSG00000031090	0,634722966	Nadsyn1	NAD synthetase 1	Upregulated
ENSMUSG00000031595	0,73085944	Pdgfrl	Platelet-derived growth factor receptor-like	Upregulated
ENSMUSG00000031750	0,632920751	Il34	Interleukin 34	Upregulated
ENSMUSG00000031778	0,795094444	Cx3cl1	Chemokine (C-X3-C motif) ligand 1	Upregulated
ENSMUSG00000032192	0,603259978	Gnb5	Guanine nucleotide binding protein (G protein), beta 5	Upregulated
ENSMUSG00000032377	0,749931001	Plscr4	Phospholipid scramblase 4	Upregulated
ENSMUSG00000032596	0,76468563	Uba7	Ubiquitin-like modifier activating enzyme 7	Upregulated
ENSMUSG00000032661	0,722618488	Oas3	2'-5' oligoadenylate synthetase 3	Upregulated
ENSMUSG00000033355	0,855232399	Rtp4	Receptor transporter protein 4	Upregulated
ENSMUSG00000034171	0,746892403	Faah	Fatty acid amide hydrolase	Upregulated
ENSMUSG00000034394	0,638254164	Lif	Leukemia inhibitory factor	Upregulated

ENSMUSG00000034459	1,698733509	Ifit1	Interferon-induced protein with tetratricopeptide repeats 1	Upregulated
ENSMUSG00000034675	0,605966812	Dbn1	Drebrin 1	Upregulated
ENSMUSG00000034855	1,143860272	Cxcl10	Chemokine (C-X-C motif) ligand 10	Upregulated
ENSMUSG00000034917	0,650775828	Tjp3	Tight junction protein 3	Upregulated
ENSMUSG00000035042	1,357138648	Ccl2	Chemokine (C-C motif) ligand 5	Upregulated
ENSMUSG00000035385	0,752402312	Ccl5	Chemokine (C-C motif) ligand 2	Upregulated
ENSMUSG00000035692	0,926093418	Isg15	ISG15 ubiquitin-like modifier	Upregulated
ENSMUSG00000036534	0,63450423	Slc38a7	Solute carrier family 38, member 7	Upregulated
ENSMUSG00000036995	0,597592667	Asap3	ArfGAP with SH3 domain, ankyrin repeat and PH domain 3	Upregulated
ENSMUSG00000037032	0,749181694	Apbb1	Amyloid beta precursor protein-binding, family B, member 1	Upregulated
ENSMUSG00000037035	0,740001008	Inhbb	Inhibin beta-B	Upregulated
ENSMUSG00000037235	0,627916782	Mxd4	Max dimerization protein 4	Upregulated
ENSMUSG00000037348	0,586268127	Paqr7	Progestin and adipoQ receptor family member VII	Upregulated
ENSMUSG00000037921	0,891919519	Ddx60	DEAD (Asp-Glu-Ala-Asp) box polypeptide 60	Upregulated
ENSMUSG00000038067	0,919139188	Csf3	Colony stimulating factor 3 (granulocyte)	Upregulated
ENSMUSG00000038178	0,730688402	Slc43a2	Solute carrier family 43, member 2	Upregulated
ENSMUSG00000038375	0,780351025	Trp53inp2	Transformation related protein 53 inducible nuclear protein 2	Upregulated
ENSMUSG00000038418	1,171852899	Egr1	Early growth response 1	Upregulated
ENSMUSG00000038521	0,594587477	C1s1	Complement component 1, s subcomponent 1	Upregulated
ENSMUSG00000038797	0,658414246	Zscan2	Zinc finger and SCAN domain containing 2	Upregulated
ENSMUSG00000039253	0,712743294	Fn3krp	Fructosamine 3 kinase related protein	Upregulated
ENSMUSG00000039865	0,667939345	Slc44a3	Solute carrier family 44, member 3	Upregulated
ENSMUSG00000039934	0,851513283	Gsap	Gamma-secretase activating protein	Upregulated
ENSMUSG00000040253	0,714131382	Gbp7	Guanylate binding protein 7	Upregulated
ENSMUSG00000040264	1,248796236	Gbp5	Guanylate binding protein 5	Upregulated
ENSMUSG00000040483	0,942431545	Xaf1	XIAP associated factor 1	Upregulated
ENSMUSG00000042099	0,678123588	Kank3	KN motif and ankyrin repeat domains 3	Upregulated
ENSMUSG00000042115	0,741056926	Klhdc8a	Kelch domain containing 8A	Upregulated
ENSMUSG00000042793	0,704398264	Lgr6	Leucine-rich repeat-containing G protein-coupled receptor 6	Upregulated

ENSMUSG00000045027	0,790735475	Prss22	Protease, serine 22	Upregulated
ENSMUSG00000045136	0,797216057	Tubb2b	Tubulin, beta 2B class IIB	Upregulated
ENSMUSG00000046447	0,591614961	Camk2n1	calcium/calmodulin-dependent protein kinase II inhibitor 1	Upregulated
ENSMUSG00000046814	0,745389063	Gchfr	GTP cyclohydrolase I feedback regulator	Upregulated
ENSMUSG00000047180	0,783767654	Neurl3	E3 ubiquitin-protein ligase NEURL3	Upregulated
ENSMUSG00000048779	0,805419043	P2ry6	Pyrimidinergic receptor P2Y, G-protein coupled, 6	Upregulated
ENSMUSG00000050578	0,679310911	Mmp13	Matrix metallopeptidase 13	Upregulated
ENSMUSG00000050957	0,641328669	Insl6	Insulin-like peptide INSL6	Upregulated
ENSMUSG00000052837	0,588551617	Junb	Transcription factor jun-B (MyD21)	Upregulated
ENSMUSG00000053835	0,617680574	H2-T24	Histocompatibility 2, T region locus 24	Upregulated
ENSMUSG00000054728	0,880911846	Phactr1	Phosphatase and actin regulator 1	Upregulated
ENSMUSG00000057137	0,780833214	Tmem140	Transmembrane protein 140	Upregulated
ENSMUSG00000057346	0,689168756	Apol9a	Apolipoprotein L 9a	Upregulated
ENSMUSG00000057969	0,83558487	Sema3b	Sema domain, immunoglobulin domain , semaphorin 3B	Upregulated
ENSMUSG00000058317	0,585891078	Ube2e2	Ubiquitin-conjugating enzyme E2	Upregulated
ENSMUSG00000059824	0,617039635	Dbp	D site albumin promoter binding protein	Upregulated
ENSMUSG00000064215	0,853256696	Ifi27	Interferon, alpha-inducible protein 27	Upregulated
ENSMUSG00000066026	0,589271788	Dhrs3	Dehydrogenase/reductase (SDR family) member 3	Upregulated
ENSMUSG00000066720	1,052486963	Cldn9	Claudin-9	Upregulated
ENSMUSG00000067212	0,587810502	H2-T23	Histocompatibility 2, T region locus 23	Upregulated
ENSMUSG00000068245	0,745782588	Phf11	PHD finger protein 11D	Upregulated
ENSMUSG00000068246	0,618452895	Apol9b	Apolipoprotein L 9b	Upregulated
ENSMUSG00000069874	0,698026095	Irgm2	Immunity-related GTPase family M member 2	Upregulated
ENSMUSG00000071656	0,616926408	Lrrn4cl	LRRN4 C-terminal like	Upregulated
ENSMUSG00000072214	0,650129549	Sept5	Septin 5	Upregulated
ENSMUSG00000072620	0,899368447	Slfn2	Schlafen 2	Upregulated
ENSMUSG00000074896	1,681198521	Ifit3	Interferon-induced protein with tetratricopeptide repeats 3	Upregulated
ENSMUSG00000078485	0,762214433	Plekhn1	Pleckstrin homology domain 1	Upregulated
ENSMUSG00000078771	0,997055051	Evi2a	Ecotropic viral integration site 2a	Upregulated

ENSMUSG00000078853	0,760398059	Igtp	Interferon gamma induced GTPase	Upregulated
ENSMUSG00000078920	0,696113103	Ifi47	Interferon gamma inducible protein 47	Upregulated
ENSMUSG00000079057	0,723439716	Cyp4v2	Cytochrome P450, family 4, subfamily v, polypeptide 3	Upregulated
ENSMUSG00000079363	0,746288686	Gbp4	Guanylate binding protein 4	Upregulated
ENSMUSG00000090877	0,886772542	Hspa1b	Heat shock protein 1B	Upregulated
ENSMUSG00000001995	-0,621712434	Sipa1l2	*Signal-induced proliferation-associated 1 like 2*	Downregulated
ENSMUSG00000019647	-1,177313293	Sema6a	*Sema domain, semaphorin 6A*	Downregulated
ENSMUSG00000019966	-0,60615575	Kitlg	*Kit ligand (Hematopoietic growth factor KL)*	Downregulated
ENSMUSG00000019982	-0,602906949	Myb	*Myeloblastosis oncogene*	Downregulated
ENSMUSG00000020142	-0,754747688	Slc1a4	*Solute carrier family 1, member 4*	Downregulated
ENSMUSG00000020256	-0,917190067	Aldh1l2	*Aldehyde dehydrogenase 1 family, member L2*	Downregulated
ENSMUSG00000020303	-0,599775563	Stc2	*Stanniocalcin 2*	Downregulated
ENSMUSG00000020307	-0,588684762	Cdc34	*Cell division cycle 34*	Downregulated
ENSMUSG00000020527	-0,648726032	Myo19	*Myosin XIX*	Downregulated
ENSMUSG00000021285	-0,651169286	Ppp1r13b	*Protein phosphatase 1, regulatory (inhibitor) subunit 13B*	Downregulated
ENSMUSG00000022802	-1,070705436	Lmln	*Leishmanolysin-like (metallopeptidase M8 family)*	Downregulated
ENSMUSG00000023067	-0,79681941	Cdkn1a	*Cyclin-dependent kinase inhibitor 1A (P21)*	Downregulated
ENSMUSG00000024841	-0,602801535	Eif1ad	*Eukaryotic translation initiation factor 1A domain containing*	Downregulated
ENSMUSG00000025185	-0,862351743	Loxl4	*Lysyl oxidase-like 4*	Downregulated
ENSMUSG00000025507	-0,591302412	Pidd1	*p53 induced death domain protein 1*	Downregulated
ENSMUSG00000027313	-0,993213094	Chac1	*ChaC, cation transport regulator 1*	Downregulated
ENSMUSG00000027737	-0,707625183	Slc7a11	*Solute carrier family 7, member 11*	Downregulated
ENSMUSG00000028212	-0,77856716	Ccne2	*Cyclin E2*	Downregulated
ENSMUSG00000028261	-0,884785106	Ndufaf4	*NADH dehydrogenase 1 alpha subcomplex, assembly factor 4*	Downregulated
ENSMUSG00000028514	-0,598251182	Usp24	*Ubiquitin specific peptidase 24*	Downregulated
ENSMUSG00000028893	-1,070912388	Sesn2	*Sestrin 2*	Downregulated
ENSMUSG00000029468	-1,477892772	P2rx7	*Purinergic receptor P2X, ligand-gated ion channel, 7*	Downregulated
ENSMUSG00000029752	-0,759012739	Asns	*Asparagine synthetase*	Downregulated
ENSMUSG00000031482	-0,6686371	Slc25a15	*Mitochondrial carrier ornithine transporter, member 15*	Downregulated

ENSMUSG00000031700	-0,681569629	Gpt2	*Glutamic pyruvate transaminase 2*	Downregulated
ENSMUSG00000031960	-0,607600916	Aars	*Alanyl-tRNA synthetase*	Downregulated
ENSMUSG00000032252	-0,659551316	Glce	*Glucuronyl C5-epimerase*	Downregulated
ENSMUSG00000032332	-1,076961446	Col12a1	*collagen, type XII, alpha 1*	Downregulated
ENSMUSG00000034875	-0,599337936	Nudt19	*nudix (nucleoside diphosphate linked moiety X)-type motif 19*	Downregulated
ENSMUSG00000035049	-0,683305313	Rrp12	*Ribosomal RNA processing 12 homolog (S. cerevisiae)*	Downregulated
ENSMUSG00000035834	-0,599419993	Polr3g	*Polymerase (RNA) III (DNA directed) polypeptide G*	Downregulated
ENSMUSG00000036875	-0,743971095	Dna2	*DNA replication helicase 2 homolog*	Downregulated
ENSMUSG00000038412	-0,686192679	Higd1a	*HIG1 domain family, member 1A*	Downregulated
ENSMUSG00000038539	-0,776267518	Atf5	*Cyclic AMP-dependent transcription factor AT*	Downregulated
ENSMUSG00000038550	-1,159135192	Ciart	*Circadian associated repressor of transcription*	Downregulated
ENSMUSG00000038658	-0,629736387	Ric1	*RAB6A GEF complex partner 1*	Downregulated
ENSMUSG00000039742	-1,272596764	Fam71f1	*Family with sequence similarity 71, member F1*	Downregulated
ENSMUSG00000040010	-0,595230426	Slc7a5	*solute carrier family 7, member 5*	Downregulated
ENSMUSG00000040447	-0,712142899	Spns2	*Spinster homolog 2*	Downregulated
ENSMUSG00000040565	-0,648190829	Btaf1	*B-TFIID transcription factor-associated*	Downregulated
ENSMUSG00000041328	-0,593533179	Pcf11	*Cleavage and polyadenylation factor subunit homolog*	Downregulated
ENSMUSG00000041762	-0,875003481	Gpr155	*G protein-coupled receptor 155*	Downregulated
ENSMUSG00000041920	-0,686498919	Slc16a6	*Solute carrier family 16, member 6*	Downregulated
ENSMUSG00000043415	-0,799086136	Otud1	*OTU domain containing 1*	Downregulated
ENSMUSG00000044165	-0,64558483	Bcl2l15	*BCLl2-like 15*	Downregulated
ENSMUSG00000044934	-0,610960072	Znf367	*Zinc finger protein 367*	Downregulated
ENSMUSG00000048058	-0,703177395	Ldlrad3	*Low density lipoprotein receptor class A domain containing 3*	Downregulated
ENSMUSG00000050244	-0,758522972	Heatr1	*HEAT repeat containing 1*	Downregulated
ENSMUSG00000054893	-1,984636828	Znf667	*Zinc finger protein 667*	Downregulated
ENSMUSG00000058006	-0,79786437	Mdn1	*Midasin homolog (yeast)*	Downregulated
ENSMUSG00000062232	-0,652214941	Rapgef2	*Rap guanine nucleotide exchange factor (GEF) 2*	Downregulated
ENSMUSG00000062345	-1,382718682	Serpinb2	*Serine (or cysteine) peptidase inhibitor, clade B, member 2*	Downregulated
ENSMUSG00000062421	-0,663726453	Arf2	*ADP-ribosylation factor 2*	Downregulated

ENSMUSG00000062785	-0,918061308	Kcnc3	*Potassium voltage gated channel, member 3*	Downregulated
ENSMUSG00000066595	-0,621210216	Mfsd7b	*Major facilitator superfamily domain containing 7B*	Downregulated
ENSMUSG00000071470	-0,774421222	Ccnb1ip1	*Cyclin B1 interacting protein 1*	Downregulated
ENSMUSG00000074221	-0,607387157	Zfp568	*Zinc finger protein 568*	Downregulated
ENSMUSG00000087141	-1,17990572	Plcxd2	*phosphatidylinositol-specific phospholipase C, X domain*	Downregulated
